# Porous Surface Films With Tunable Morphologies and Hydrophobic Properties Based on Block Copolymer Under the Effects of Thermal Annealing

**DOI:** 10.3389/fchem.2019.00181

**Published:** 2019-03-28

**Authors:** Guadalupe del C. Pizarro, Oscar G. Marambio, Manuel Jeria-Orell, Diego P. Oyarzún, Rudy Martin-Trasanco, Julio Sánchez

**Affiliations:** ^1^Departamento de Química, Universidad Tecnológica Metropolitana, Santiago, Chile; ^2^Facultad de Ciencias Exactas, Center of Applied Nanosciences, Universidad Andrés Bello, Santiago, Chile; ^3^Departamento de Ciencias del Ambiente, Facultad de Química y Biología, Universidad de Santiago de Chile, Santiago, Chile

**Keywords:** controlled radical polymerization, self-assembly diblock copolymers, thermal behavior, morphological surface characteristic, hydrophobic surfaces, self-assembled copolymers, porous films, surface morphology

## Abstract

The fabrication of porous-structured polymer films with patterned surface structures has recently attracted increased interest within the material science field. In this work, a series of microstructure scale patterned polymer films were obtained via breath figure methods (BF) with hydrophobic surface films based on self-assembled diblock copolymers by atom-transfer radical polymerization (ATRP). The surface characteristics and morphological properties, pore size, roughness, thickness, and wettability of the block copolymer films was studied in response to variation of the hydrophilic co-monomer structures. A significant improvement of the quality and order of the hydrophobic films was observed in response to thermal annealing and a consequent optimization of the assembly process.

## Introduction

Block copolymers provide the perfect platform for fabricating novel microstructured materials for advanced technologies, for example bio-related materials (Lai et al., 2011; Pizarro et al., 2016). Additionally, these materials are considered very attractive for use in the synthesis of functional microstructures (Kim et al., 2007; Oz et al., 2008; Wen et al., 2008; Tobis et al., 2010; Zhang et al., 2010; Yao et al., 2011). Within such fields, polymer domain size, separation, and degree of order are used to identify the impact of the different process parameters in order to achieve optimization of the self-assembly process (Guarini et al., 2002). The morphology and domain size are generally controlled by adjusting individual block lengths, while copolymer molecular weights define the absolute template dimensions (Shirtcliffe et al., 2011; Cardoso et al., 2015; Hong et al., 2016). Moreover, recent studies have demonstrated that is possible to obtain superhydrophobic surfaces by tuning the roughness and morphology of the co-polymer block surface (Wu et al., 2016). Several superhydrophobic surfaces have been obtained on hydrophilic materials by inducing an adequate roughness (Hosono et al., 2005; Zhu et al., 2006; Cao et al., 2007; Karlsson et al., 2010; Celia et al., 2013). The literature reveals the great interest for fundamental research and practical applications in these topics (Lai et al., 2011; Shirtcliffe et al., 2011; Yao et al., 2011; Kumar et al., 2015).

In this work we report the preparation of block copolymer films with varying structures. This was achieved by inducing changes to the structure and morphology of hydrophilic co-monomers via template processing and thermal annealing processes. It is well known that the properties of film surfaces strongly depend on uniformity at the micro/nanometer-scale (i.e., the structural domains) and on the hierarchical structures constituted by these domains. Both factors often play the main role within the desired application. The micro-structured porous films prepared herein may constitute new materials for potential application in biomedicine; for example as support for cell growth with biological purposes. Additionally, the hydrophilic domains composed by carboxylic acid groups should serve as 3D-scaffolds for further chemical modifications which would hold further potential within such fields as catalysis and gas/chemical sensors (Phillip et al., 2010; Nunes et al., 2011; Hilke et al., 2013; Baettig et al., 2017).

In this article, we specifically report a series of experiments in which we systematically varied template processing parameters in order to understand their influence on the self-assembly process. Additionally, we investigate the effect of thermal annealing on the control the uniformity of the films, maintaining the thickness of the films and molecular weight practically constant. The block copolymers containing different block units are expected to produce self-assembled microstructures with tunable morphology giving porous and rough surface films. Subsequently, the wettability of the porous polymer films was also investigated.

The control over the copolymer structure is crucially important to the preparation of porous structured films. For this reason, atom-transfer radical polymerization (ATRP) was used. The ATRP technique is more versatile than other polymerization methods such as synthetic route based on anionic and cationic polymerizations. For the latter, rigorous operation conditions are required and the reaction is limited to certain types of monomers. On the other hand, ATRP is very attractive because of its tolerance to impurities, compatibility with a wide range of monomers, and relatively mild polymerization conditions (Wang and Matyjaszewski, 1995; Coca et al., 1998; Huang et al., 2012).

The block copolymers were prepared using a ATRP technique based on polystyrene (PS) (first block) and the hydrophilic co-monomers dimethyl allylmalonate (DMM), dimethyl itaconate (DMI), and tert-butyl acrylate (tBA) forming the second block. The block copolymers were characterized using size exclusion chromatography (SEC), FT-IR, Raman, and ^1^H-NMR spectroscopy. Thermal analyses were conducted by TGA and DSC. Subsequently, the porous polymer films were prepared on glass substrates under controlled experimental conditions. The surface morphologies of the polymer porous films were characterized with scanning electron microscopy (SEM) and atomic force microcopy (AFM), the hydrophilicity of the surface was observed via static water contact angle (WCA). Finally, the film thickness was obtained via ellipsometry.

## Experimental Details

### Materials

Copper(I) bromide (CuBr, 98%) was supplied by Sigma-Aldrich and was further purified with repeated stirring overnight in acetone at room temperature. The solid was washed with ethanol and diethylether prior to drying at 50°C under vacuum for 24 h. Benzoyl peroxide (BPO, 75% remainder water), 2,2-bipyridine (BPy, 99%) and tetrabutylammonium fluoride (1.0 N in THF) were purchased from Sigma-Aldrich and were used as received. Styrene (Sigma-Aldrich Chemicals) was washed with 5% NaOH aqueous solution and was then distilled under reduced pressure prior to use. All other reagents and solvents were analytical grade and were utilized as received: Dimethyl allylmalonate (DMM), dimethyl itaconate (DMI), and tert-butyl acrylate (tBA) (Sigma-Aldrich, 99%).

### Instrumentation and Equipments

^1^H-NMR spectra were recorded in solution using a Bruker AC 250 (Bruker, Karlsruhe, Germany) spectrometer at room temperature with deuterated dimethylsulfoxide (DMSO-d_6_, 99.8%) and deuterated chloroform (CDCl_3_), as solvent. FT-IR spectra were performed using a Bruker Vector 22 (Bruker Optics GmbH, Inc., Ettlingen, Germany). The molecular weights [number average (*M*_n_) and weight average (*M*_w_)], and the molecular weight distribution (polydispersity, *M*_w_/*M*_n_) of the polymers were determined by size exclusion chromatography (SEC) using a Shimatzu LC 20 instrument equipped with RI detectors with DMF as the solvent (flow rate: 1.0 mL/min). The structural and vibrational properties of the copolymer films were characterized using Raman spectroscopy with a LabRam 010 instrument from ISA equipped with a 5.5 mW, 632.8 nm He-Ne laser without a filter. The Raman microscope used back-scattering geometry, where the incident beam is linearly polarized at a 500:1 ratio. The objective of the microscope lens was an Olympus Mplan 100x (numerical aperture 0.9). The optical properties were examined by UV-Vis absorption and fluorescence emission spectra. The absorption spectra of the films were recorded at 25°C between 250 and 700 nm using a Perkin Elmer Lambda 35 spectrophotometer. Thermal studies were recorded using a Star System 1 thermogravimetric analyzer (TGA) at a heating rate of 10°C/min. The morphological properties of the self-assembled block copolymers were examined by SEM, and AFM. The SEM was a model LEO 1420VP with a 100 μA beam current and a working distance of 12–14 mm. The microscope was operated at high vacuum (system vacuum ~10^−6^ mbar and chamber 10^−3^ mbar). The surface morphology of the copolymer porous films was characterized by AFM using Nanonics MultiView MV1000. In AFM measurements, n-type silicon cantilevers (f_1/4_ 37.2*kHz*; *k*_1/4_ 0.01–0.60 N/m; tip radius 10 nm) in contact mode or optical fiber cantilevers were employed. Statistical analysis of the images obtained was performed using image processing software Gwyddion 2.37. The powder polymer was dissolved in THF and cast on glass slides using a spin coating technique with rotation velocity ramps of 500 rpm for approximately 10 s and 1,600 rpm for ~10 s. A KW-4A spin coater (Chemat Scientific), coupled with an oil free vacuum pump (Rocker Chemker 410), was utilized for deposit of the copolymer solutions on glass substrate. Contact angle measurements were performed in a Ramé-hart model 250 (p/n 250-U1) standard goniometer/tensiometer using a sessile drop on drop method over the solid substrate. A Multi-angle laser ellipsometer model SE 400adv (SENTECH Instrument GmbH) was used to perform optical measurements; He–Ne laser (λ = 633 nm) in PCSA null configuration which was used to determine the film thickness. The angle of incidence of the laser beam was 60.5° with respect to the normal sample.

### Synthesis and Characterization of Macroinitiator

The synthesis of the PS was carried out according to the literature (Beers et al., 1999; Teodorescu et al., 2000; Min et al., 2005). The styrene and the catalyst system [BPO]:[CuBr]:[BPy] at a mole ratio of 100:1:1/2 were added into a polymerization flask under nitrogen gas. The resulting polymer was precipitated into methanol, and then the product was dried at 60°C under vacuum to a constant weight (yield: ~80%). The macroinitiator was obtained with a narrow polydispersity index (*D* = 1.13). Experimental data of the PS-Br is presented in Table 1. The ^1^H-NMR spectra (δ ppm) of PS showed the signals at 1.42 [2H, -CH_2_ from the backbone]; 1.86-[1H, > CH–Ar side chain, respectively, from S] and 6.60 [2H, m, H–Ar], at 7.34 [3H, s, H–Ar]. The FT-IR spectra exhibited characteristic absorption bands at 3,026 cm^−1^ [υ(CH, CH_2_ and CH aromatic)]; 2,921 cm^−1^ [υ(CH, CH_2_)]; 1,943–1,721 cm^−1^ [υ(aromatic overtone)]; 1,600 cm^−1^ (tension C-C, aromatic ring) and 1,448 cm^−1^ [υ(flexion -C-H, CH_2_)]; 756 and 697 cm^−1^ [υ(flexion mono substitute aromatic ring)].

**Table 1:** Experimental conditions and results of amphiphilic block copolymers.

**Samples**	***Mn*** **^a^**_**(theo)**_/10^**3**^ g mol^**−1**^	***M*n** **^b^**_**(SEC)**_/10^**3**^ g mol^**−1**^	**Ð = Mw/Mn**
PS	10.4	11.7	1.13
PS-*b*-PAA	13.9	14.8	1.20
PS-*b*-PIA	14.9	16.3	1.10
PS-*b*-PAMA	14.0	14.1	1.22

a *Theoretical number average molar mass*.

b *Mn was determined from SEC in THF*.

### Synthesis of the Block Copolymers

**Scheme 1:**
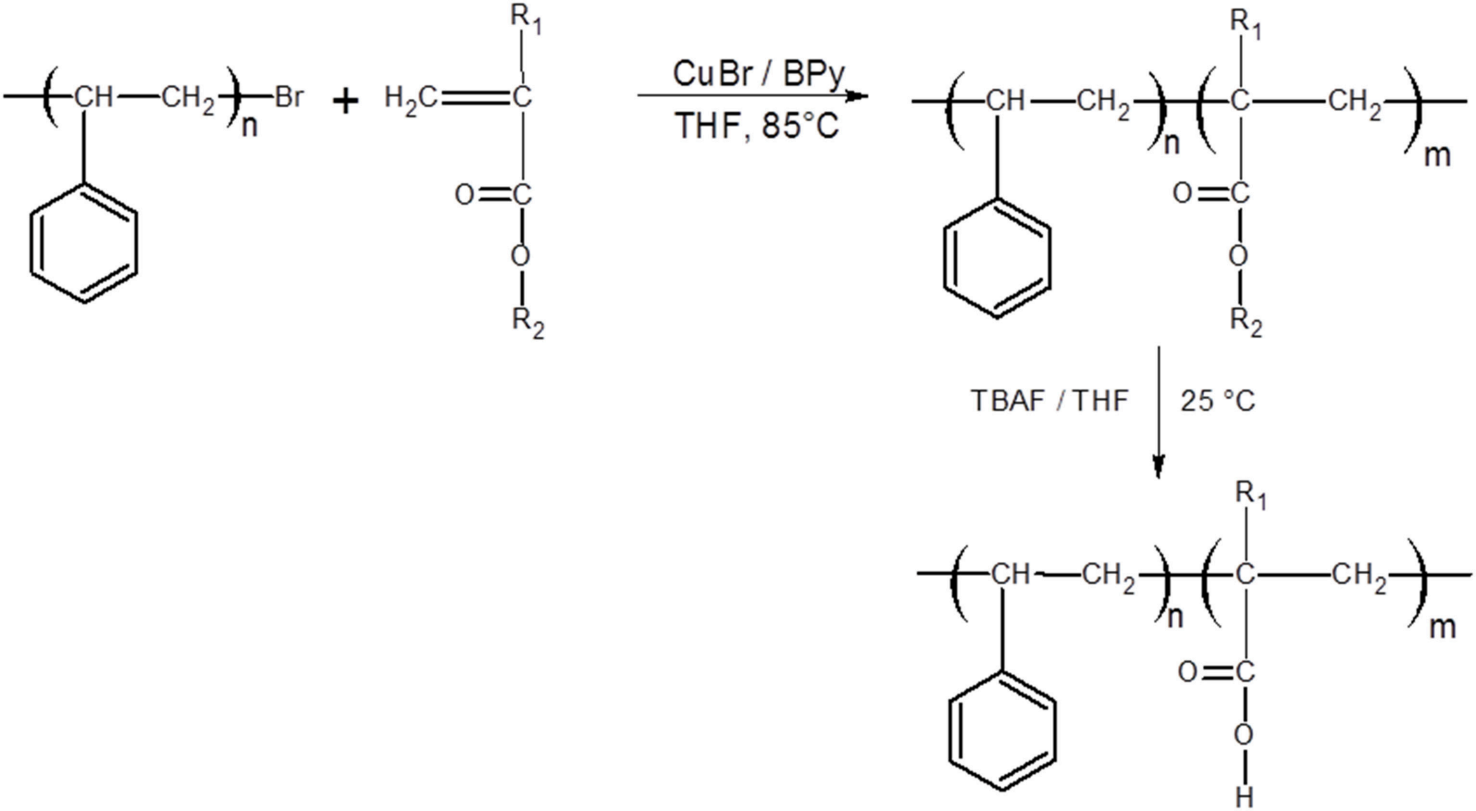
Structures of the self-assembled block copolymers: PS-*b*-PAA; PS-***b***-PIA; and PS-***b***-PAMA were obtained from its precursors by hydrolysis procedure, respectively.

The amphiphilic block copolymers based on PS with the acrylic acid (AA), itaconic acid (IA), and allylmalonic acid (AMA) blocks were obtained via ATRP using CuBr/BPy as the catalyst system with the co-monomers DMM/DMI/tBA in a molar ratio of 1/1/2/100 forming first the precursors poly(styrene-*b*-tert-butylmethacrylate) PS-*b*-PtBA; poly(styrene-*b*-dimethylallylmalonate) PS-*b*-PDMM and poly(styrene-*b*-dimethylitaconate) PS-*b*-PDMI and then the amphiphilic block copolymers PS-b-PAA, PS-b-PIA. Subsequently PS-b-PAMA were obtained, with a molecular weight approximately between 14,000 and 16,500 Da, see Table 1 and Scheme 1. This was performed in accordance with similar reported procedures (Coca et al., 1998; Hamley, 1998; Beers et al., 1999; Teodorescu et al., 2000; Min et al., 2005; Pizarro et al., 2013, 2015).

#### Procedure

The synthesis of PS-*b*-PDMM block copolymer was carried out with the PS-Br/CuBr/BPy/co-monomers in a mole ratio of 1/1/2/100 (Coca et al., 1998; Pizarro et al., 2013). First, 0.1581 mmol of macroinitiator, assuming the molar mass of PS is *Mn* = 11,701 Da, was added to a polymerization flask and dissolved in 3 mL of THF. Subsequently, 15.81 mmol of DMM monomer and 0.3162 mmol of ligand BPy were then added under vigorous stirring at room temperature. Then, the mixture was purged with liquid nitrogen. While stirring at 25°C for 20 min, 0.158 mmol of CuBr was added to the system and placed in an oil bath at 85°C for 4 h. After reaction, the resulting PS-*b*-PDMM, was dissolved in THF and repeatedly precipitated into methanol and then dried at 60°C under vacuum up to constant weight to obtain the final product (yield: ~60%). Similar procedures were carried out for the PS-*b*-PtBA and PS-*b*-PDMI. A series of block copolymers, with narrow dispersity (*D* = 1.11–1.23), were obtained by acidic hydrolysis of the alkyl ester linkages, obtaining the block copolymers with assembling behavior, see Table 1 and Scheme 1. Similar polydispersity indexes were reported elsewhere (Ashford et al., 1999; Davis et al., 2000; Wang et al., 2000; Ke et al., 2010).

### Preparation of the Samples for Morphological Study

Approximately 3 mg of polymer was stirred in 1 mL of solvent (THF). The resultant solution was cast onto a glass substrate. The porous films were usually obtained within 30 min due to the high solvent volatility. Subsequently, the samples were heated at 120°C for 2 h.

## Results and Discussion

The block copolymerization based on polystyrene with tert-butyl acrylate (tBA), has been reported previously (Coca et al., 1998; Beers et al., 1999; Teodorescu et al., 2000; Min et al., 2005; Pizarro et al., 2013, 2015; Yu et al., 2016), however block copolymers with dimethyl allylmalonate (DMM), and dimethyl itaconate (DMI) as specific monomers were used. To the best of our knowledge, block copolymers with DMM and DMI monomers have not yet been reported. The PS-*b*-PtBA was taken as comparative model.

### Characterization of the Block Copolymers

**Figure 1:**
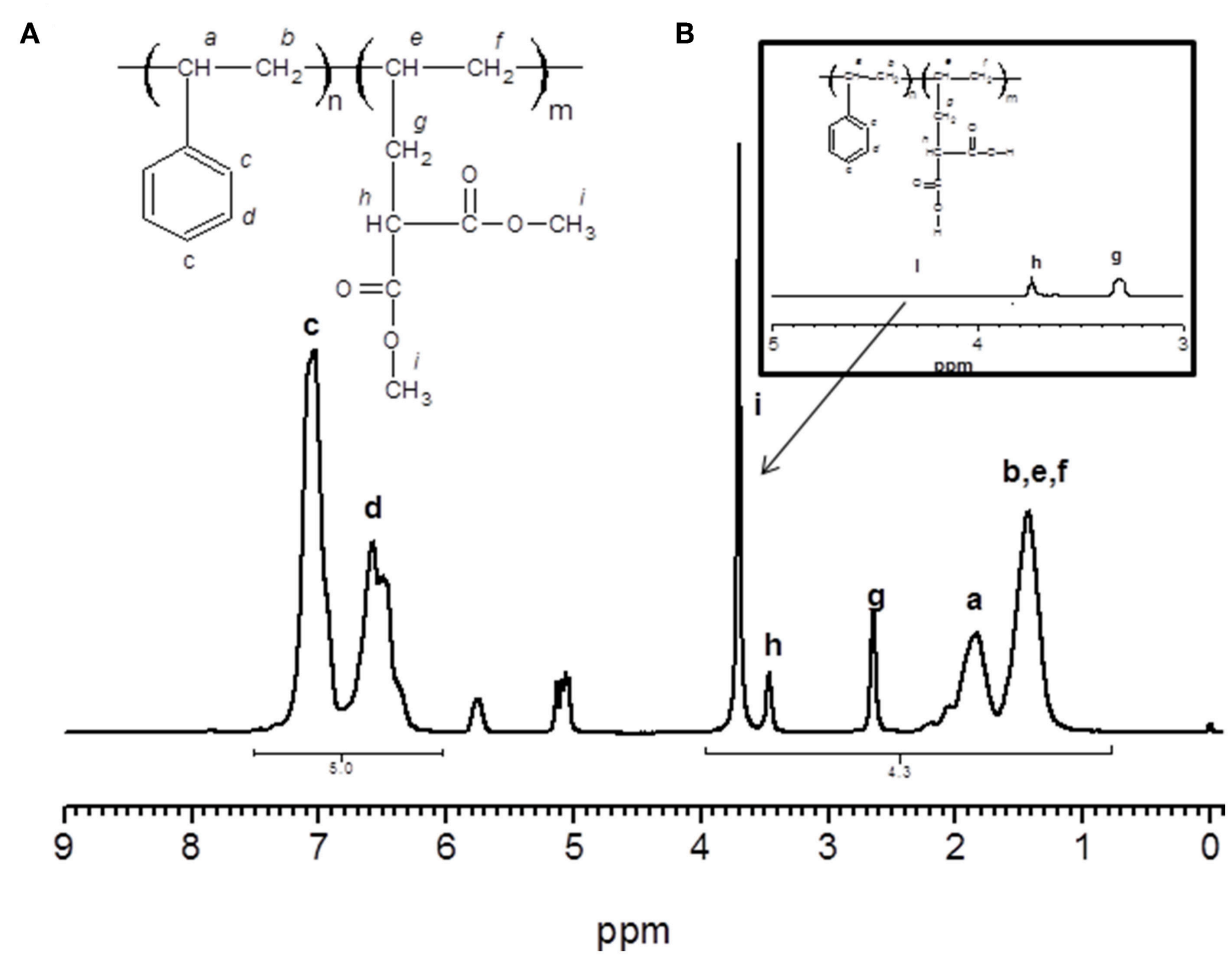
**(A,B)** ^1^H-NMR spectra of PS-*b*-DMM and PS-*b*-PAMA (CDCl_3_).

The block copolymers were soluble in chloroform, acetone, DMF, and DMSO. The ^1^H NMR spectra (400 Hz, CDCl_3_, δ ppm) for PS-*b*-PDMM block copolymer (as an example), clearly shows resonance signals at (δ, ppm): **7.20** [3H, s (wide), H–Ar]; **6.60** [2H, m, H–Ar]; **3.75** [6H, s, -O-CH_3_]; **3.60** [1H, s, >CH–]; **2.60** [2H, s, –CH_2_-]; **1.90** [1H, s (wide), >CH–Ar]; **1.42** [5H, s (wide), >CH-. –CH_2_-], see Figure 1. The ^1^H NMR spectra (400 Hz, CDCl_3_, δ ppm) for the PS-*b*-PAMA block copolymer (as an example), shows the same signals, except the signal of the hydrolyzed block copolymer which shows a decrease in signal intensity at 3.5–3.6 ppm which corresponds to the percent hydrolysis of the alkyl ester—indicating the formation of -COOH groups. The hydrolysis procedure was realized by treatment with tetrabutylammonium fluoride [TBAF, (C_4_H_9_)_4_NF] as described in the literature (Hamley, 1998), yield in weight, ~80%.

### Raman Analysis of the Copolymers

**Figure 2:**
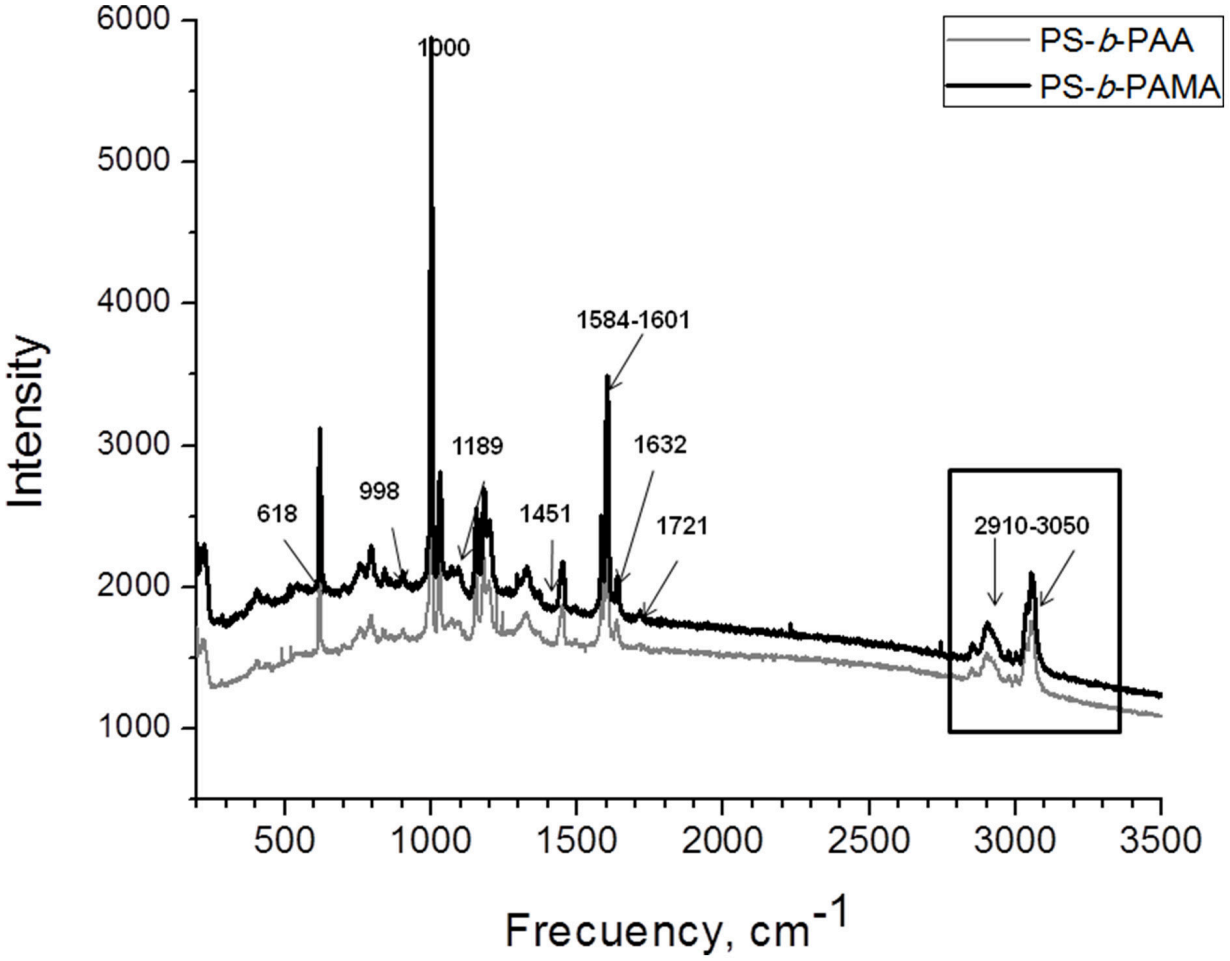
Raman spectra of PS*-b-*PAA and PS*-b-*AMA block copolymers.

Raman spectra (see Figure 2) exhibited a strong peak at 2,910–3,050 cm^−1^ assigned to the aromatic protons (C–H stretching in plane bending), and a band of low intensity at 1,721 cm^−1^ assigned to C=O (stretching). The band at 1,451 cm^−1^ was assigned to alkenes (–CH bending). The band at 1,189 cm^−1^ was assigned to C–O stretching of ester groups. A strong signal at 998 cm^−1^ was assigned to C–C aromatic (stretching), and the band at 618 cm^−1^ was assigned to C–H aromatic (stretching out of plane in opposite direction).

### Thermal Studies and Optical Properties

**Figure 3:**
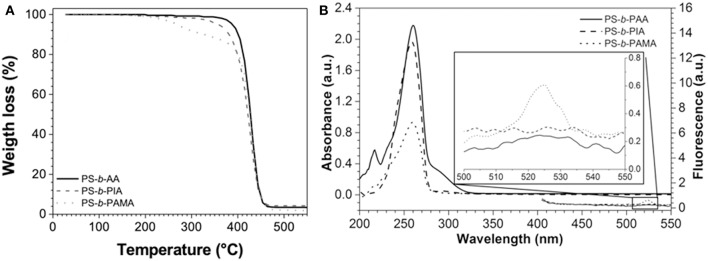
**(A)** TGA thermograms of PS-b-PAA, and PS-b-PIA and PS-b-PAMA block copolymers; **(B)** Optical properties of PS-b-PAA, PS-b-PIA and PS-b-PAMA block copolymers.

TGA was employed to evaluate the effect of hydrophilic block on the thermal decomposition temperature (TDT) of the diblock copolymers. The TGA curves in the Figure 3A exhibit three decomposition curves in one-step; for PS-*b*-PAA and PS-*b*-PIA. The resulting block copolymers have an extrapolated thermal decomposition temperature (TDT) from 380 to 388°C, when decomposition of the backbone chain occurs; except for the case of the PS-*b*-PAMA block copolymers which exhibit a weight loss approximately of 10% at 400°C, see Figure 3A.

The optical properties of the material exhibited one characteristic band at 260 nm (see Figure 3B). This intense absorption band was attributed to the π-π^*^ transition due to electron delocalization in the aromatic ring (inter-molecular transfer change) of the first block (PS). The fluorescence emission spectra of the PS-*b*-PAMA block copolymers exhibited a weak band at 525 nm (green luminescence emission). The π-π^*^ transition is assigned to the interactions between neighboring –COOH functional groups in these polar co-monomers and also the distance between –COOH groups. Transparent materials with high UV absorption can be prepared using these block copolymer as polymeric matrices.

### Morphological Characterization of the Film

**Figure 4:**
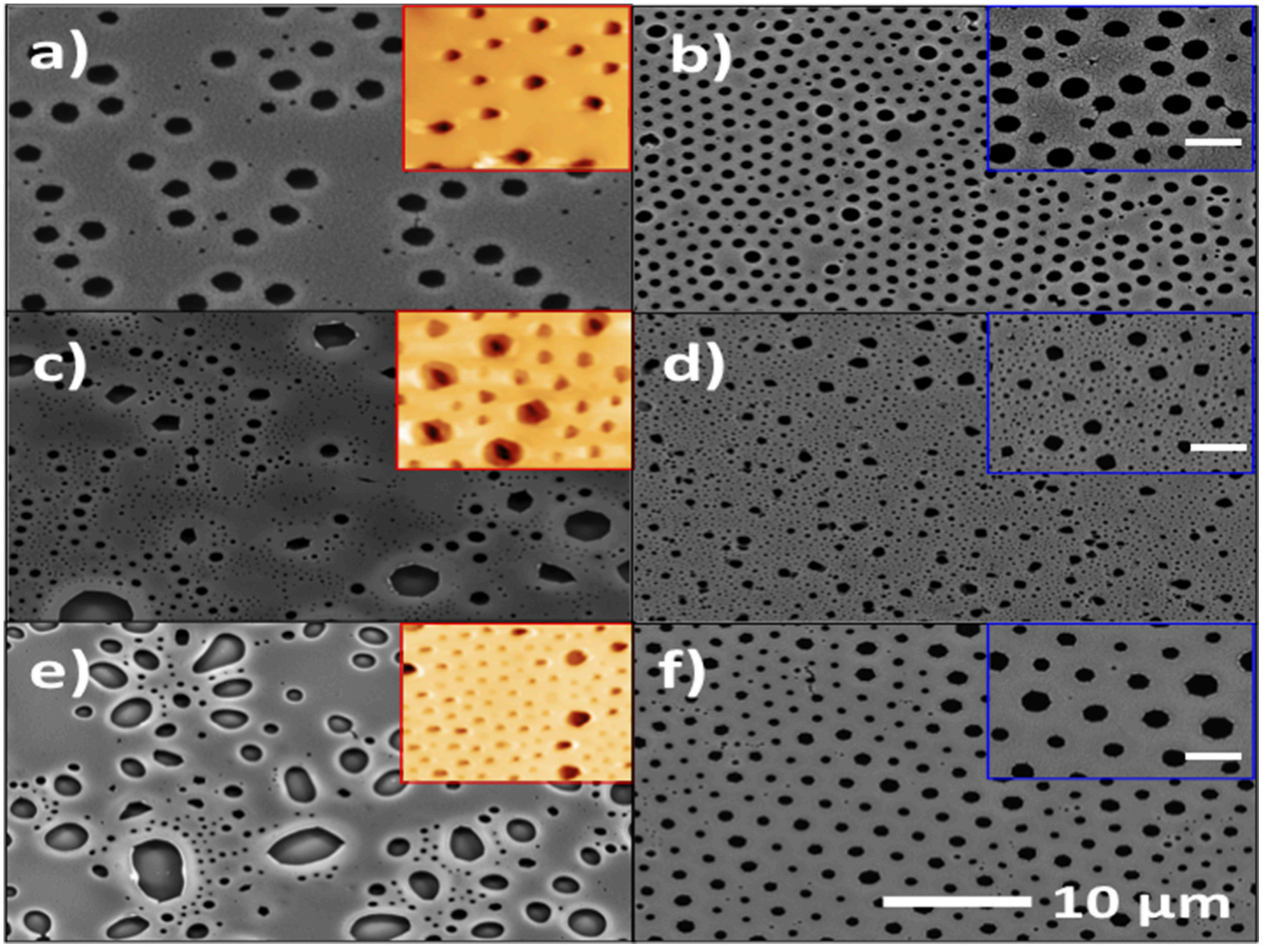
SEM micrographs of the surfaces of PS-*b*-PAA **(a)**, PS-*b*-PIA **(c)**, PS-*b*-PAMA **(e)** before annealing, and PS-*b*-PAA **(b)**, PS-*b*-PIA **(d)**, PS-*b*-PAMA **(f)** after annealing. Inset of **(a,c,e)** are the corresponding AFM micrographs. Inset of **(b,d,f)** are the corresponding magnified micrographs (scale bar = 2 μm).

In this work, all samples were compared considering similar molecular weight (14,000–16,500 Da) and similar film thicknesses. The morphological images were obtained by SEM and AFM using the constant force method (see Figures 4a–f). The films showed a large distribution of elongated pore sizes with varying pore spacing. The PS-*b*-AA block copolymer samples displayed smaller pore diameter size (from ~1.0 to 2.5 μm) than the PS-*b*-PIA samples which exhibited pore diameter size ranging between ~2.5 and 4.0 μm. In addition, the PS-*b*-PAMA block copolymer exhibited a differing surface morphology; a rough structure with straight cylinder-like morphology was observed for this case (see Figures 4a,c,e). This different behavior was attributed mainly to the choice of monomer, where the phase separation can occur at slower rates for PS-*b*-AMA and PS-*b*-PIA block copolymer systems. The AFM images showed that the block copolymers tend to self-assemble due to their amphiphilic characteristics, displaying a rough surface with a pore diameter size ranging from ~1.0 to 4.0 μm and depth from ~ 2.1 to 2.2 μm. The difference in the morphologies is attributed to several factors that affect the phase separation such as the nature of monomers, copolymer composition, molecular size, and molecular configuration (Hamley, 1998). The results of the film thickness and depth were obtained by ellipsometry, see Table 2.

**Table 2:** Thickness and pore diameter and pore depth of thin porous polymer films.

**Samples**	**Thickness of the films(nm)**	**Pore diameter (nm)**	**Pore depth (nm)**
PS-*block*-PAA^*^	2,391 ± 20	1,236 ± 55	2,210 ± 61
PS-*block*-PIA^*^	2,362 ± 16	2,849 ± 50	2,050 ± 40
PS-*block*-PAMA^*^	2,224 ± 25	4,100 ± 140	2,150 ± 50

* *C = 3 mg/mL (THF); thickness of the films was obtained using ellipsometry, pore depth, and pore diameter size (maximum values) were obtained by AFM*.

### Morphological Properties Under Annealing Process

Subsequently, for the PS-*b*-PIA and PS-*b*-PAMA films, the effect of thermal annealing on the self-assembly process was explored due to its low degree of porous uniformity encountered. The film thickness for the block copolymers was controlled by maintaining constant spin speeds and solution concentrations. The block copolymer films were annealed at 120°C for 2 h. An important effect on the resulting morphological quality was thus observed which resulted in a high degree of uniformity of the pores (see Figure 4). This change was attributed to the experimental procedure being performed at temperatures above the glass-transition temperature (Tg = 98.3°C and 94.3°C), which favored the speed of spontaneous self-assembly for PS-*b*-PIA and PS-*b*-PAMA block copolymers which improved the quality and order in the films. These results indicate that porous-film quality was improved under the described thermal annealing process (see Figures 4b,d,f). The pore size diameter values were from 1.0 to 4.0 μm. It also was possible to observe smaller pore sizes (1 μm) for PS-*b*-PAA block copolymer samples compared to the PS-*b*-PIA samples which exhibited pore diameter size (approximately from 1.0 to 1.5 μm). In contrast, the PS-*b*-PAMA block copolymer, which exhibited a different behavior under these experimental conditions; showed a porous structured morphology but with dispersed pore sizes (approx. from 1.5 to 4.0 μm). Hence, the porous films with thermal annealing process possess smaller pore diameter size than the films without annealing. This outcome contrasts results previously reported from Pizarro et al. (2013).

### Contact Angle on the Polymeric Film With and Without Annealing Process

The surface wettability surface of materials is known to be mainly dependent on the geometrical structure and the chemical composition, with consequent practical applications (Wan et al., 2012; Sasmal et al., 2014; Cheng et al., 2015; Yu et al., 2016). A direct expression of the wettability of a surface is the water contact angle (WCA), which a water drop is placed on the film surface for WCA measurements. The roughness and porosity of the surface films observed using SEM are expected to affect the contact angle values.

**Figure 5:**
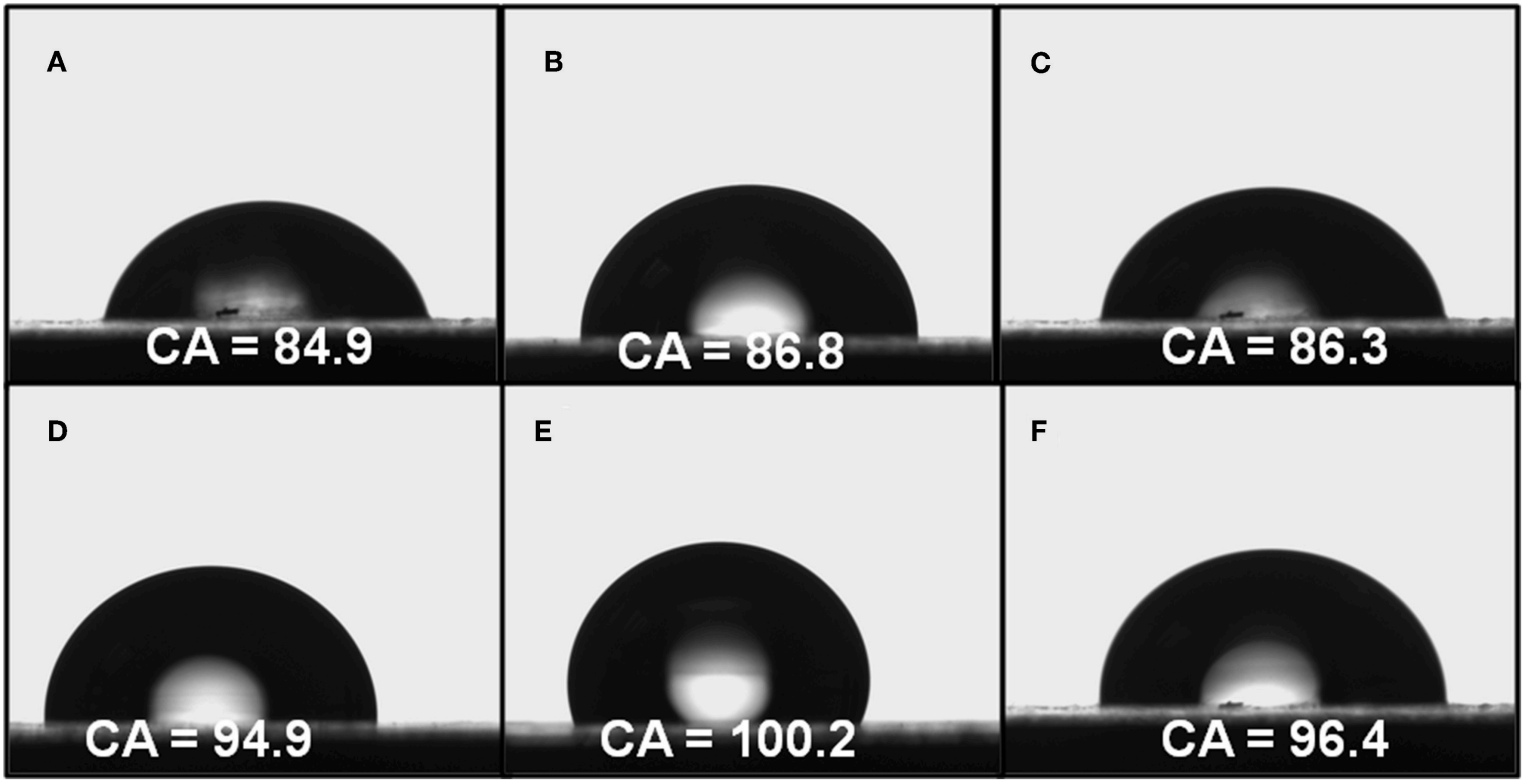
Water contact angle (WCA) images of the surfaces of without and with annealing **(A,D)** PS-*b*-PAA, **(B,E)** PS-*b*-PIA, **(C,F)** PS-*b*-PAMA.

In this case, when the films were annealed at 120°C (for 2 h) [above the glass transition temperature (92–98°C)], the water droplet, can penetrate into the cavities of surface (pore depth approximately of 230 nm). It is the high roughness which increases the contact area which then increases the liquid–solid interaction. For contact angles <90°, the surface is conventionally described as hydrophilic, if the contact angle varies between 90° and 150°, the surface is hydrophobic, and if WAC is >150°, the surface is conventionally described as a superhydrophobic surface. Before thermal annealing, the PS-*b*-PAA film showed the lower WCA whilst PS-*b*-PIA and PS-*b*-PAMA showed similar values. The WCA values of the polymer's films submitted to the annealing process, increases, except for PS that remains constant. It is expected that the thermal annealing provokes a partial rearrangement of the polymer chains and therefore the probability of self-assembling of copolymers carrying –COOH groups. This self-assembling leads to an increase of the hydrophobicity since the no longer availability of this carboxylic acid groups to interact with water. Based on this WCA values range, the values varied from 96.4° to 103.2° for the polymer films, according with these results is possible to conclude that the films exhibited good hydrophobicy properties (see Figure 5 and Table 3). This behavior was attributed to the annealing process that produces a change in the self-assembly of the films and in the order and formation of the pores. These changes promote hydrophobicity on the surface by reducing the surface tension, while its carboxylic acid functions is anchored the polymer film onto glass substrate and probably also into cavities of the pores. The increases in the contact angle value suggest additional hydrogen bonding interactions between the majority carboxylic acid groups on the surface and into the pores that influence the wettability of the films pre-annealing. These porous films exhibited a WAC >90°, which indicates its hydrophobic characteristics (see Table 3).

**Table 3:** Water contact angle (WCA) values of the porous polymer films without and with annealing(^*^).

**Films**	**Left**	**Right**	**Mean**
PS	104.6°	106.5°	105.6°
PS^*^	**106.1°**	**108.5°**	**107.3°**
PS-*b*-PAA	84.9°	82.8°	83.6°
PS-*b*-PAA^*^	**94.9°**	**97.8°**	**96.4°**
PS-*b*-PIA	86.8°	90.1°	89.1°
PS-*b*-PIA^*^	**100.2**	**106.9**	**103.5**
PS-*b*-PAMA	86.3°	90.8°	87.3°
PS-*b*-PAMA^*^	**96.4°**	**98.5°**	**97.5°**

*Contact angle of the glass substrate = 51.9°*.

## Conclusions

This work has presented the synthesis of a series of diblock copolymers which were used for the preparation of hydrophobic structured polymer films. The films displayed surface structures patterned at microstructure scale. The block copolymers showed high thermal stability temperatures (TDT) of approximately 380°C. The optical properties of the material exhibited one characteristic absorption band at 260 nm. This intense absorption band was attributed to the π-π^*^ transition due to electron delocalization in the aromatic ring (inter-molecular transfer change) of the first block (PS). The SEM and AFM images showed the block copolymers tended to form a rough and porous surface films with a pore size diameter ranging between ~1.0 and 4.0 μm. This variation was due to their amphiphilic characteristics under experimental annealing conditions. Post annealing procedures demonstrated that the quality, porous uniformity and degree of order were improved; due to the spontaneously self-assembled diblock copolymers being favored. This suggests that the variation of the hydrophilic block (the number of –COOH in the polar co-monomers) can produce these differences in the surface films, moreover increasing or decreasing the intermolecular interactions of the functional groups of the matrices (-COOH groups) due to mutual orientation and distance between –COOH groups. The results indicate that the phase behavior of the block copolymers is predominately determined by the PS block with hydrophobic characteristics, which was predominant in the formation of the films.

## Author Contributions

GP: design, synthesis and characterization of monomers, discussion of experiments, and paper writing; OM: contribution in FT-IR and NMR spectroscopy analysis and discussion of the corresponding results; MJ-O: analysis and discussion of fluorescence results; DO: TGA analysis; RM-T: analysis of SEM Micrographs and paper writing; JS: analysis and discussion on films wettability and paper writing.

### Conflict of Interest Statement

The authors declare that the research was conducted in the absence of any commercial or financial relationships that could be construed as a potential conflict of interest.

## References

[B1] Ashford E. J. Naldi V. O'Dell R. Billingham N. C. Armes S. P. (1999). First example of the atom transfer radical polymerisation of an acidic monomer: direct synthesis of methacrylic acid copolymers in aqueous media. Chem. Commun. 33, 1285–1286. 10.1039/a903773j

[B2] Baettig J. Oh J. Bang J. Khan A. (2017). Sequential coating of nanopores with charged polymers: a general approach for controlling pore properties of self-assembled block copolymer membranes. Macromol. Res. 25, 1091–1099. 10.1007/s13233-017-5142-5

[B3] Beers K. L. Boo S. Gaynor S. G. Matyjaszewski K. (1999). Atom transfer radical polymerization of 2-hydroxyethyl methacrylate. Macromolecules 32, 5772–5776. 10.1021/ma990176p

[B4] Cao L. Hu H. A. Gao D. (2007). Design and fabrication of micro-textures for inducing a superhydrophobic behavior on hydrophilic materials. Langmuir 23, 4310–4314. 10.1021/la063572r 17371061

[B5] Cardoso V. F. Botelho G. Lanceros-Méndez S. (2015). Nonsolvent induced phase separation preparation of poly(vinylidene fluoride- co -chlorotrifluoroethylene) membranes with tailored morphology, piezoelectric phase content and mechanical properties. Mater. Des. 88, 390–397. 10.1016/j.matdes.2015.09.018

[B6] Celia E. Darmanin T. Taffin de Givenchy E. Amigoni S. Guittard F. (2013). Recent advances in designing superhydrophobic surfaces. J. Colloid Interface Sci. 402, 1–18. 10.1016/j.jcis.2013.03.041 23647693

[B7] Cheng Z. Wang J. Lai H. Du Y. Hou R. Li C.. (2015). PH-controllable on-demand oil/water separation on the switchable superhydrophobic/superhydrophilic and underwater low-adhesive superoleophobic copper mesh film. Langmuir 31, 1393–1399. 10.1021/la503676a 25563562

[B8] Coca S. Jasieczek C. B. Beers K. L. Matyjaszewski K. (1998). Polymerization of acrylates by atom transfer radical polymerization. Homopolymerization of 2-hydroxyethyl acrylate. J. Polym. Sci. Part A Polym. Chem. 36, 1417–1424.

[B9] Davis K. A. Charleux B. Matyjaszewski K. (2000). Preparation of block copolymers of polystyrene and poly (t-butyl acrylate) of various molecular weights and architectures by atom transfer radical polymerization. J. Polym. Sci. Part A Polym. Chem. 38, 2274–2283. 10.1002/(SICI)1099-0518(20000615)38:12<2274::AID-POLA170>3.0.CO;2-I

[B10] Guarini K. W. Black C. T. Yeung S. H. I. (2002). Optimization of diblock copolymer thin film self assembly. Adv. Mater. 14, 1290–1294. 10.1002/1521-4095(20020916)14:18<1290::AID-ADMA1290>3.0.CO;2-N

[B11] Hamley I. W. (1998). The Physics of Block Copolymers. Oxford: Oxford University.

[B12] Hilke R. Pradeep N. Madhavan P. Vainio U. Behzad A. R. Sougrat R.. (2013). Block copolymer hollow fiber membranes with catalytic activity and pH-response. ACS Appl. Mater. Interfaces 5, 7001–7006. 10.1021/am401163h 23865535

[B13] Hong Q. Ma X. Li Z. Chen F. Zhang Q. (2016). Tuning the surface hydrophobicity of honeycomb porous films fabricated by star-shaped POSS-fluorinated acrylates polymer via breath-figure-templated self-assembly. Mater. Des. 96, 1–9. 10.1016/j.matdes.2016.01.137

[B14] Hosono E. Fujihara S. Honma I. Zhou H. (2005). Superhydrophobic perpendicular nanopin film by the bottom-up process. J. Am. Chem. Soc. 127, 13458–13459. 10.1021/ja053745j 16190684

[B15] Huang B. Fan X. Wang G. Zhang Y. Huang J. (2012). Synthesis of twin-tail tadpole-shaped (cyclic polystyrene)- block-[linear poly (tert-butyl acrylate)]2 by combination of glaser coupling reaction with living anionic polymerization and atom transfer radical polymerization. J. Polym. Sci. Part A Polym. Chem. 50, 2444–2451. 10.1002/pola.26021

[B16] Karlsson M. Forsberg P. Nikolajeff F. (2010). From hydrophilic to superhydrophobic: Fabrication of micrometer-sized nail-head-shaped pillars in diamond. Langmuir 26, 889–893. 10.1021/la902361c 19775135

[B17] Ke B.-B. Wan L.-S. Zhang W.-X. Xu Z.-K. (2010). Controlled synthesis of linear and comb-like glycopolymers for preparation of honeycomb-patterned films. Polymer 51, 2168–2176. 10.1016/j.polymer.2010.03.021

[B18] Kim S. G. Lim J. Y. Sung J. H. Choi H. J. Seo Y. (2007). Emulsion polymerized polyaniline synthesized with dodecylbenzene-sulfonic acid and its electrorheological characteristics: temperature effect. Polymer 48, 6622–6631. 10.1016/j.polymer.2007.09.013

[B19] Kumar D. Wu X. Fu Q. Ho J. W. C. Kanhere P. D. Li L. (2015). Hydrophobic sol–gel coatings based on polydimethylsiloxane for self-cleaning applications. Mater. Des. 86, 855–862. 10.1016/j.matdes.2015.07.174

[B20] Lai Y.-K. Chen Z. Lin C.-J. (2011). Recent progress on the superhydrophobic surfaces with special adhesion: from natural to biomimetic to functional. J. Nanoeng. Nanomanufactur. 1, 18–34. 10.1166/jnan.2011.1007

[B21] Min K. Gao H. Matyjaszewski K. (2005). Preparation of homopolymers and block copolymers in miniemulsion by ATRP Using Activators Generated by Electron Transfer (AGET). J. Am. Chem. Soc. 127, 3825–3830. 10.1021/ja0429364 15771517

[B22] Nunes S. P. Karunakaran M. Pradeep N. Behzad A. R. Hooghan B. Sougrat R.. (2011). From micelle supramolecular assemblies in selective solvents to isoporous membranes. Langmuir 27, 10184–10190. 10.1021/la201439p 21710987

[B23] Oz K. Yavuz M. Yilmaz H. Unal H. I. Sari B. (2008). Electrorheological properties and creep behavior of polyindole/poly(vinyl acetate) composite suspensions. J. Mater. Sci. 43, 1451–1459. 10.1007/s10853-007-2319-x

[B24] Phillip W. A. O'Neill B. Rodwogin M. Hillmyer M. A. Cussler E. L. (2010). Self-assembled block copolymer thin films as water filtration membranes. ACS Appl. Mater. Interfaces 2, 847–853. 10.1021/am900882t 20356290

[B25] Pizarro G. d. C. Jeria-Orell M. Marambio O. G. Olea A. F. Valdés D. T. Geckeler K. E. (2013). Synthesis of functional poly(styrene)- block -(methyl methacrylate/methacrylic acid) by homogeneous reverse atom transfer radical polymerization: spherical nanoparticles, thermal behavior, self-aggregation, and morphological properties. J. Appl. Polym. Sci. 129, 2076–2085. 10.1002/app.38923

[B26] Pizarro G. d. C. Marambio O. G. Jeria-Orell M. González-Henríquez C. M. Sarabia-Vallejos M. Geckeler K. E. (2015). Effect of annealing and UV-radiation time over micropore architecture of self-assembled block copolymer thin film. Express Polym. Lett. 9, 525–535. 10.3144/expresspolymlett.2015.50

[B27] Pizarro G. del C. Marambio O. G. Jeria-Orell M. Oyarzún D. P. Geckeler K. E. (2016). Size, morphology and optical properties of ZnO nanoparticles prepared under the influence of honeycomb-porous poly[(2-hydroxyethylmethacrylate)m- block -poly(N -phenyl maleimide)n] copolymer films. Mater. Des. 111, 513–521. 10.1016/j.matdes.2016.09.036

[B28] Sasmal A. K. Mondal C. Sinha A. K. Gauri S. S. Pal J. Aditya T. (2014). Fabrication of superhydrophobic copper surface on various substrates for roll-o ff, self-cleaning, and water/oil separation. ACS Appl. Mater. Interfaces 6, 22034–22043. 10.1021/am5072892 25419984

[B29] Shirtcliffe N. J. McHale G. Newton M. I. (2011). The superhydrophobicity of polymer surfaces: Recent developments. J. Polym. Sci. Part B Polym. Phys. 49, 1203–1217. 10.1002/polb.22286

[B30] Teodorescu M. Gaynor S. G. Matyjaszewski K. (2000). Halide anions as ligands in iron-mediated atom transfer radical polymerization. Macromolecules 33, 2335–2339. 10.1021/ma991652e

[B31] Tobis J. Thomann Y. Tiller J. C. (2010). Synthesis and characterization of chiral and thermo responsive amphiphilic conetworks. Polymer 51, 35–45. 10.1016/j.polymer.2009.10.055

[B32] Wan L. S. Li J. W. Ke B. B. Xu Z. K. (2012). Ordered microporous membranes templated by breath figures for size-selective separation. J. Am. Chem. Soc. 134, 95–98. 10.1021/ja2092745 22142340

[B33] Wang J.-S. Matyjaszewski K. (1995). Controlled/“living” radical polymerization. atom transfer radical polymerization in the presence of transition-metal complexes. J. Am. Chem. Soc. 117, 5614–5615. 10.1021/ja00125a035

[B34] Wang X. S. Jackson R. A. Armes S. P. (2000). Facile synthesis of acidic copolymers via atom transfer radical polymerization in aqueous media at ambient temperature. Macromolecules 33, 255–257. 10.1021/ma000671h

[B35] Wen W. Huang X. Sheng P. (2008). Electrorheological fluids: structures and mechanisms. Soft Matter 4, 200–210. 10.1039/B710948M 32907231

[B36] Wu X. Fu Q. Kumar D. Ho J. W. C. Kanhere P. Zhou H. (2016). Mechanically robust superhydrophobic and superoleophobic coatings derived by sol-gel method. Mater. Des. 89, 1302–1309. 10.1016/j.matdes.2015.10.053

[B37] Yao X. Song Y. Jiang L. (2011). Applications of bio-inspired special wettable surfaces. Adv. Mater. 23, 719–734. 10.1002/adma.201002689 21287632

[B38] Yu H. Liu J. Fan X. Yan W. Han L. Han J. (2016). Bionic micro-nano-bump-structures with a good self-cleaning property: the growth of ZnO nanoarrays modified by polystyrene spheres. Mater. Chem. Phys. 170, 52–61. 10.1016/j.matchemphys.2015.12.018

[B39] Zhang W. He J. Liu Z. Ni P. Zhu X. (2010). Biocompatible and pH-responsive triblock copolymer mPEG- b -PCL- b -PDMAEMA: Synthesis, self-assembly, and application. J. Polym. Sci. Part A Polym. Chem. 48, 1079–1091. 10.1002/pola.23863

[B40] Zhu M. Zuo W. Yu H. Yang W. Chen Y. (2006). Superhydrophobic surface directly created by electrospinning based on hydrophilic material. J. Mater. Sci. 41, 3793–3797. 10.1007/s10853-005-5910-z

